# Fatty Acid Derivatives Isolated from the Oil of *Persea americana* (Avocado) Protects against Neomycin-Induced Hair Cell Damage

**DOI:** 10.3390/plants10010171

**Published:** 2021-01-18

**Authors:** SeonJu Park, Seo Yule Jeong, Youn Hee Nam, Jun Hyung Park, Isabel Rodriguez, Ji Heon Shim, Tamanna Yasmin, Hee Jae Kwak, Youngse Oh, Mira Oh, Kye Wan Lee, Jung Suk Lee, Do Hoon Kim, Yu Hwa Park, In Seok Moon, Se-Young Choung, Kwang Won Jeong, Bin Na Hong, Seung Hyun Kim, Tong Ho Kang

**Affiliations:** 1 Chuncheon Center, Korea Basic Science Institute (KBSI), Chuncheon 24341, Korea; sjp19@kbsi.re.kr; 2 Department of Oriental Medicine Biotechnology, College of Life Sciences and Graduate School of Biotechnology, Kyung Hee University, Gyeonggi 17104, Korea; tjdbf26@gmail.com (S.Y.J.); 01030084217@hanmail.net (Y.H.N.); isabelula3r@gmail.com (I.R.); jee1015235@gmail.com (J.H.S.); pm.tamanna.yasmin@gmail.com (T.Y.); habina22@hanmail.net (B.N.H.); 3 Yonsei Institute of Pharmaceutical Sciences, College of Pharmacy, Yonsei University, Incheon 21983, Korea; kayskaron@naver.com (J.H.P.); moon3685@naver.com (H.J.K.); oys9300@naver.com (Y.O.); purunmei2002@naver.com (M.O.); 4 R&D Center, Dongkook Pharm. Co. Ltd., Gyeonggi 16229, Korea; lkw1@dkpharm.co.kr (K.W.L.); ljs@dkpharm.co.kr (J.S.L.); kdh2@dkpharm.co.kr (D.H.K.); pyh@dkpharm.co.kr (Y.H.P.); 5 Department of Otorhinolaryngology, Yonsei University College of Medicine, Seoul 03722, Korea; ISMOONMD@yuhs.ac; 6 Department of Preventive Pharmacy and Toxicology, College of Pharmacy, Kyung Hee University, Seoul 02453, Korea; sychoung@khu.ac.kr; 7 Gachon Institute of Pharmaceutical Sciences, College of Pharmacy, Gachon University, Incheon 21936, Korea; kwjeong@gachon.ac.kr

**Keywords:** avocado oil, fatty acids, hearing loss, zebrafish, hair cell

## Abstract

Avocado oil is beneficial to human health and has been reported to have beneficial effects on sensorineural hearing loss (SNHL). However, the compounds in avocado oil that affect SNHL have not been identified. In this study, we identified 20 compounds from avocado oil, including two new and 18 known fatty acid derivatives, using extensive spectroscopic analysis. The efficacy of the isolated compounds for improving SNHL was investigated in an ototoxic zebrafish model. The two new compounds, namely (2*R*,4*R*,6*Z*)-1,2,4-trihydroxynonadec-6-ene and (2*R*,4*R*)-1,2,4-trihydroxyheptadecadi-14,16-ene (compounds 1 and 2), as well as compounds 7, 9, 14, 17 and 19 showed significant improvement in damaged hair cells in toxic zebrafish. These results led to the conclusion that compounds from avocado oil as well as oil itself have a regenerative effect on damaged otic hair cells in ototoxic zebrafish.

## 1. Introduction

Sensorineural hearing loss (SNHL) is a major disease that may be genetic or acquired as a consequence of disease, ototoxic drugs or chemicals, noise, or trauma, among other causes [1,2,3]. Around 466 million people in world have disabling hearing loss, and this number will be estimated to be over 900 million by 2050 [4]. Therefore, it is important to focus on preventing hearing loss. In a continuing study to identify phytochemicals that protect against auditory hair cell damage, we investigated the protective effects of compounds from avocado leaves on SNHL in vitro [5].

Avocado (*Persea americana* Mill.) is a fruit tree that is indigenous to tropical and subtropical regions [6]. Due to its functional properties and nutritional value, interest in the study of various parts of avocado tree such as its fruit, leaves and oil has increased. Avocado contains approximately 60% oil [7]. Avocado oil is renowned for its healing and renewing effects and reduced inflammation during the wound healing process [8]. In addition, it is a functional food that composed of a high content of unsaturated fatty acids especially oleic acid and linoleic acid (about 50% and 10% in total fatty acid), which are beneficial to human health. Among various unsaturated fatty acids, omega-3 fatty acids have previously demonstrated efficacy in preventing age-related hearing loss [9,10]. We previously reported the efficacy of avocado oil on sensorineural hearing loss in vitro and in vivo [2]. However, the compounds in avocado oil that improve SNHL have not been identified. Therefore, we aimed to isolate compounds from avocado oil and confirm their efficacy for improving hearing loss in an ototoxic zebrafish model. Thus, in a continuing project to identify phytochemicals that improve auditory hair cell function, phytochemical investigation and the biological evaluation of avocado oil led to isolation of two new compounds and 18 known fatty acid derivatives. The protecting effects of the isolated compounds against neomycin-induced hearing loss was investigated in a zebrafish model.

## 2. Results and Discussion

### 2.1. Structure Elucidation

**Figure 1:**
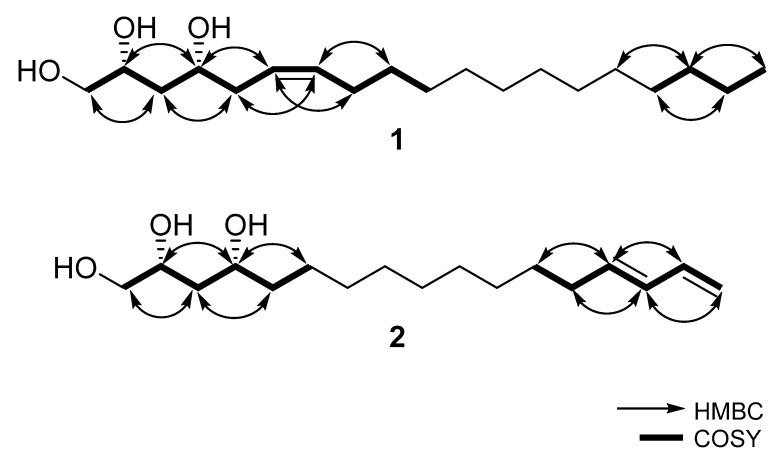
The key HMBC and COSY correlations of **1** and **2**.

Compound **1** was isolated as a colorless oil. The molecular formula was determined to be C_19_H_38_O_3_ on the basis of HR–ESI–MS pseudo-ion at *m*/*z* 297.2775 [M + H−H_2_O]^+^ (calcd for C_19_H_37_O_2_, 297.2788). The ^1^H-NMR spectrum of **1** was similar to that of (2*R*,4*R*,6*E*)-1,2,4-trihydroxynonadec-6-ene [11]. There were three hydroxy proton signals at *δ*_H_ 3.52 (2H, dd, *J* = 5.3, 14.3 Hz), 3.81 (1H, dt, *J* = 4.1, 8.2 Hz) and 3.85 (1H, ddt, *J* = 2.4, 4.4, 9.2 Hz) (Table 1). In **1**, two *cis* olefinic protons at *δ*_H_ 5.38 and 5.39 (each 1H, overlapped) replaced the two *trans* olefinic protons at 5.45 and 5.50 (each 1H, dt, *J* = 6, 15 Hz). A double bond that is located at C-6 and C-7 was verified from the COSY (Figure 1), which exhibited correlations between H-5 (*δ*_H_ 2.82) and H-4 (*δ*_H_ 3.81), H-6 (*δ*_H_ 5.39); H-7 (*δ*_H_ 5.38) and H-6 (*δ*_H_ 5.39), H-8 (*δ*_H_ 2.11). The ^13^C and HSQC spectra of **1** exhibited nineteen carbon signals, including fourteen methylenes (one oxygenated), four methines (two olefinics) and one methyl signal. Unlike the two *trans* olefinic carbons of (2*R*,4*R*,6*E*)-1,2,4-trihydroxynonadec-6-ene at *δ*_C_ 127.2 and 134.4, the signal difference between the two *cis* olefinic carbons of 1 at *δ*_C_ 129.1 and 130.9 is smaller. Thus, this allows the planar structure of **1** to be (6*Z*)-1,2,4-trihydroxynonadec-6-ene. Based on the NOE correlation between H-2 (*δ*_H_ 3.85) and H-4 (*δ*_H_ 3.81) and comparing the optical rotation of **1** with two possible synthesized stereoisomers of 1,2,4-trihydroxyheptadec-16-ene, the absolute configuration of 1 is 2*R*, 4*R*. The synthetic (2*R*,4*R*)-1,2,4-trihydroxyheptadec-16-ene has given negative optical rotation (${\left[\alpha \right]}_{D}^{20}$: −6.4 (c 1.1, CHCl_3_)) as compound **1**, whereas (2*S*,4*S*)-1,2,4-trihydroxyheptadec-16-ene has positive value, ${\left[\alpha \right]}_{D}^{20}$: +6.0 (c 1.0, CHCl_3_) [12]. Thus, the structure of **1** is (2*R*,4*R*,6*Z*)-1,2,4-trihydroxynonadec-6-ene.

Compound **2** was isolated as a colorless oil and its molecular formula was confirmed to be C_17_H_32_O_3_ by the HR–ESI–MS ion at *m*/*z* 267.2314 [M + H − H_2_O]^+^ (calcd for C_17_H_31_O_2_, 267.2324). The ^1^H NMR spectrum showed three major regions: a large number of methylenes from *δ*_H_ 1.20 to 1.60, several oxygenated protons from *δ*_H_ 3.50 to 3.80, and two double bonds from *δ*_H_ 4.90 to 6.30. The ^13^C and HSQC spectra of **1** exhibited seventeen carbon signals, including twelve methylenes (one olefinic, one oxygenated) and five methines (three olefinic) signals. The ^1^H-NMR spectrum of **2** exhibited similar ^1^H signals to that of compound **1** except for the additional double bond. The most downfield ^1^H signal (*δ*_H_ 6.32, H-16) displayed a typical exomethylene double bond. *J* values of 17.0 and 10.3 Hz were observed for *trans* and *cis* protons (H-17*_trans_* and H-17*_cis_*), respectively. H-17*_trans_* (*δ*_H_ 5.07) exhibited a dd pattern with *J* values of 1.8 and 17.0 Hz due to H-16 and H-17*_cis_*, while H-17*_cis_* (*δ*_H_ 4.93) with a ddt pattern displayed *J* values of 1.8 and 10.3 Hz for H-16 and H-17*_trans_*. Additionally, another double bond was located next to the terminal double bond, which was determined by correlations between H-16 (*δ*_H_ 6.32)/H-15 (*δ*_H_ 6.03) and C-13 (*δ*c 33.6)/C-14 (*δ*c 136.2)/C-15 (*δ*c 132.4)/C-16 (*δ*c 138.7)/C-17 (*δ*c 114.9) (Figure 1). A previous study related to relative configuration was conducted by synthesizing all four stereoisomers of 1,2,4-trihydroxyheptadec-16-ene. Both compounds **2** and (2*R*,4*R*)-1,2,4-trihydroxyheptadec-16-ene exhibited negative optical rotation and thus the configurations of C-2 and C-4 were determined to be *R* [12]. Based on the evidence above, compound **2** is (2*R*,4*R*)-1,2,4-trihydroxyheptadecadi-14,16-ene.

**Figure 2:**
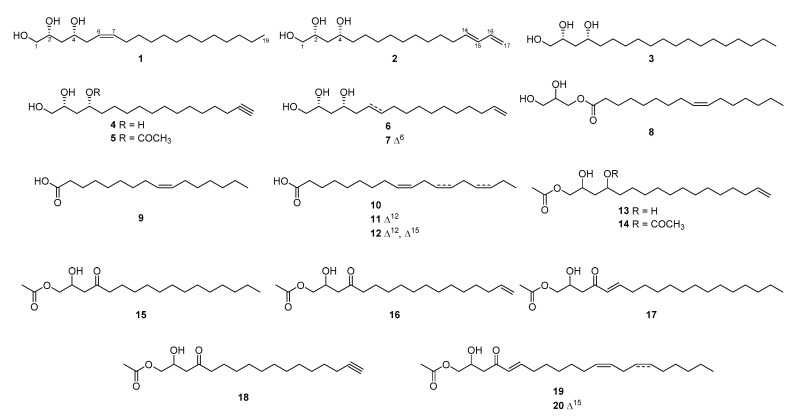
Chemical structures of compounds **1**–**20**.

By comparing the NMR and MS data with previously reported literature, known compounds were identified as (2*R*,4*R*)-1,2,4-trihydroxynonadecane, (2*R*,4*R*)-1,2,4-trihydroxyheptadec-16-yne, avocadene (**3**–**4**, **6**) [13], 4-acetoxy-1,2-dihydroxyheptadec-16-yne (**5**) [11], avocadenol A (**7**) [14], 1-palmitoleylglycerol (**8**) [15], palmitoleic acid and oleic acid (**9**–**10**) [16], linoleic acid (**11**) [17], *α*-linolenic acid (**12**) [18], avocadene acetate, 1-(acetyloxy)-2-hydroxy-4-heptadecanone, avocadenone acetate (**13**, **15**–**16**) [19], 1,4*R*-diacetoxy-2*R*-hydroxyheptadeca-16-ene (**14**) [20], persenone B (**17**) [21], avocadyne acetate (**18**) [22], (5*E*,12*Z*)-2-hydroxy-4-oxoheneicosa-5,12-dien-1-yl acetate and (5*E*,12*Z*,15*Z*)-2-hydroxy-4-oxoheneicosa-5,12,15-trien-1-yl acetate (**19**–**20**) [23] (Figure 2).

### 2.2. Otic Hair Cell Recovery

**Figure 3:**
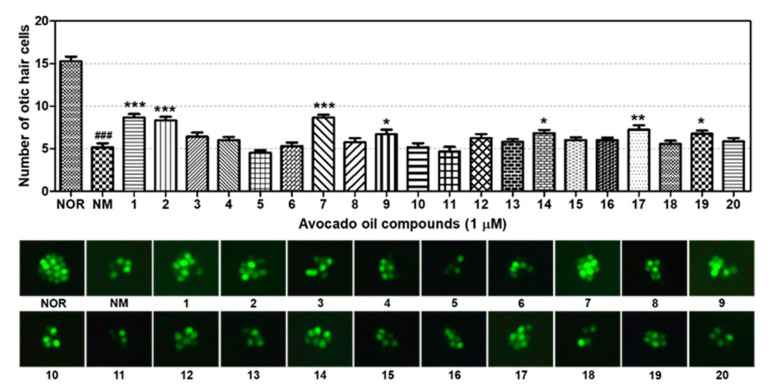
Otic hair cell recovery of compounds **1**–**20** after neomycin-induced hair cell damage. Data are presented as means ± SEM. ### *p* < 0.001 (NOR group versus NM group). * *p* < 0.05, ** *p* < 0.01 and *** *p* < 0.001 (NM versus avocado oil compounds treated groups).

The effects of compounds **1**–**20** on otic hair cell recovery were evaluated after neomycin exposure using a zebrafish model. Hair cells in zebrafish neuromast have reported apoptosis during normal turnover and after damage by aminoglycosides, similarly to the hair cells in an organ of Corti in mammals [24,25,26,27,28,29,30]. In addition, genes and pathways involved in apoptosis, neuronal development, and oxidation are highly conserved in zebrafish [31,32], which allows to study the effects of potential therapeutics, such as neurotrophic factors, antioxidants and antiapoptotic agents on hair cell loss. The hair cells within the otic (O1) neuromast were severely damaged by neomycin exposure (Figure 3). Neomycin significantly decreased the number of otic hair cells (*p* < 0.001). However, 1 µM of compound **1** (*p* < 0.001), **2** (*p* < 0.001), **7** (*p* < 0.001), and **9** (*p* = 0.04) exhibited significant hair cell recovery compared to the control. Furthermore, compounds **14** (*p* = 0.01), **17** (*p* = 0.006), and **19** (*p* = 0.01) led to significant recovery in hair cells compared to controls. In our previous study, avocado oil had a protective effect against neomycin-induced ototoxicity in sensory hair cells. These beneficial effects may be related to avocado oil compounds **1**, **2**, **7**, **9**, **14**, **17**, and **19**, which significantly increased otic hair cell recovery after neomycin-induced ototoxicity in a zebrafish model. All those compounds except **9** are the avocado-derived fatty acids. Compound **9**, palmitoleic acid, is a monounsaturated fatty acid that can be found in a variety of animal fats, vegetable oils, and marine oils. Future studies should focus on these compounds and their mechanism of action in SNHL.

## 3. Materials and Methods

### 3.1. General Experimental Procedures

The resonances for ^1^H and ^13^C were observed at 400 and 100 MHz, respectively, in an Agilent 400-MR-NMR spectrometer. Chemical shifts are expressed in parts per million in the scale relative to tetramethylsilane. Data processing was performed by the MestReNova ver. 6.0.2 program. HR–ESI–MS analyses and prep-HPLC were carried out by an AGILENT 6550 iFunnel Q-TOF LC/MS system and by an AGILENT 1200 HPLC system, respectively. Column chromatography was done on silica–gel (Kieselgel 60, 70–230 mesh and 230–400 mesh, Merck, Kenilworth, NJ, USA) or YMC RP-18 resins (30–50 μm, Fujisilisa Chemical Ltd., Kasugai Aichi, Japan). A pre-coated silica–gel 60 F254 (0.25 mm, Merck) and RP-18 F254S plates (0.25 mm, Merck) were used for thin layer chromatography (TLC).

### 3.2. Plant Material

Avocado oil that was made from whole parts of the fruit (peel, pulp and seed) was purchased from Avoplus in Morelia, Mexico, in 2018. It was manufactured by a general manufacturing process for avocado oil: grinding fully ripe fruits and undergoing centrifugal extraction. Avocado fruits were supplied by certified producers and Avoplus, the oil manufacturer, has its certification.

### 3.3. Extraction and Isolation

The oil of *P. americana* (60 g) was subjected to column chromatography (CC) separation over silica gel and eluted with a gradient of CHCl_3_: MeOH (17:1, *v*/*v*) gave four smaller fractions, AVO1 (0.4 g), AVO2 (0.3 g), AVO3 (0.8 g) and AVO4 (0.8 g) and cleansed with MeOH (AVO-M). The AVO1 fraction was performed prep-HPLC with J’sphere ODS H-80 column (250 mm × 20 mm), eluting with 78% MeCN and a flow rate of 3 mL/min to yield 15 (12.7 mg), 16 (14.1 mg), 17 (18.6 mg), 18 (1.9 mg), 19 (4.2 mg) and 20 (43.3 mg). The AVO2 fraction was also applied to prep-HPLC as conditions above with 75% MeCN to yield 11 (117.4 mg), 12 (13.2 mg), and 13 (53.2 mg), whereas the AVO4 fraction was applied to silica gel CC, eluting with CHCl_3_: MeOH (20:1, *v*/*v*) to obtain 10 (994.0 mg). The AVO-M fraction was applied to a silica gel CC with CHCl_3_: MeOH (15: 1, *v*/*v*) and three sub-fractions, AVO-M1 (1.0 g), AVO-M2 (1.3 g) and AVO-M3 (1.8 g), were obtained. The AVO-M1 fraction was purified further using a RP-18 CC, which eluted with acetone: H_2_O (4:1, *v*/*v*) to yield 8 (13.4 mg), 9 (23.2 mg), and 14 (44.1 mg). The AVO-M2 fraction was subjected to prep-HPLC as per the above conditions with 65% MeCN to yield 1 (2.4 mg), 2 (4.0 mg), and 4 (13.7 mg). Lastly, the AVO-M3 fraction was performed and RP-18 CC eluted with acetone: H_2_O (4:1, *v*/*v*) to yield 3 (23.4 mg), 5 (32.7 mg), 6 (2.3 mg), and 7 (3.2 mg).

#### 3.3.1. (2*R*,4*R*,6*Z*)-1,2,4-Trihydroxynonadec-6-ene (**1**)

Colorless oil; ${\left[\alpha \right]}_{D}^{20}$: −33.4 (c 0.02, CHCl_3_); C_19_H_38_O_3_, HR-ESI-MS *m*/*z*: 297.2775 [M + H − H_2_O]^+^ (calcd for C_19_H_37_O_2_, 297.2788); ^1^H (CD_3_OD, 600 MHz) and ^13^C NMR (CD_3_OD, 100 MHz) data can be seen in Table 1.

#### 3.3.2. (2*R*,4*R*)-1,2,4-Trihydroxyheptadecadi-14,16-ene (**2**)

Colorless oil; ${\left[\alpha \right]}_{D}^{20}$: −38.0 (c 0.02, CHCl_3_); C_17_H_32_O_3_, HR-ESI-MS *m*/*z*: 267.2314 [M + H − H_2_O]^+^ (calcd for C_17_H_31_O_2_, 267.2324); ^1^H (CD_3_OD, 600 MHz) and ^13^C NMR (CD_3_OD, 100 MHz) data can be seen in Table 1.

### 3.4. Animals

Wild-type adult zebrafish (*Danio rerio*) were cultured in a zebtec stand-alone system (1500(W) × 400(D) × 2050(H) mm, WoojungBio, Inc., Suwon, Korea). Three pairs of zebrafish were set-up overnight in a spawning box and eggs were gathered 3 h after fertilization. Embryos were cultured in 0.03% of sea salt solution (Sigma-Aldrich Co., St. Louis, MO, USA) in a petri dish under a 14 h light/10 h dark cycle in an incubator at 26.5–28.5 °C until 6 days post-fertilization (dpf) when the experiments were performed as previously described [2].

### 3.5. Ethical Statement

All zebrafish experimental procedures were performed in accordance with standard zebrafish protocols and the Animal Care and Use Committee of Kyung Hee University [KHUASP(SE)-15-10] approved the experiments.

### 3.6. Neomycin-Induced Ototoxicity in a Zebrafish Model

Wild-type zebrafish larvae were sited into a 96-well plate and treated with 100 µL of 2 µM neomycin sulfate (MB Cell Co., Seoul, Korea) for an hour to induce ototoxicity as previously described [2].

To evaluate the protective effects of compounds 1–20 on otic hair cell damage, the neomycin solution was removed and zebrafish were rinsed with 0.03% sea salt solution. The zebrafish were then treated with 100 μL of 1 μM compounds 1–20. After 8 h treatment, the zebrafish were rinsed again with 0.03% sea salt solution and stained for 30 min with 0.1% YO-PRO-1 (Fisher Scientific Inc., Hampton, MA, USA), then anesthetized with 0.04% tricaine. The otic hair cells were counted under a fluorescence microscope (Olympus 1 × 70; Olympus Co., Tokyo, Japan) after visualization. Image analysis was performed by Focus Lite software (Focus Co., Daejeon, Korea).

### 3.7. Statistical Analysis

Data were analyzed by GraphPad Prism (version 5) statistical software package (GraphPad, San Diego, CA, USA). All data are expressed as the means ± standard error of the mean. The statistical significance of the differences between groups was determined by a paired *t*-test. *p*-value of <0.05 (*), <0.01 (**) and <0.001 (***) are considered statistically significant.

## 4. Conclusions

A detailed phytochemical study of avocado oil identified two previously undescribed fatty acid derivatives, (2*R*,4*R*,6*Z*)-1,2,4-trihydroxynonadec-6-ene and (2*R*,4*R*)-1,2,4-trihydroxyheptadecadi-14,16-ene, as well as 18 known compounds. We evaluated the protective effect of these 20 compounds against neomycin-induced ototoxicity in a zebrafish model. Our results demonstrated that new compounds 1 and 2, as well as compounds 7, 9, 14, 17, and 19 protect otic hair cells from ototoxicity.

## Figures and Tables

**Table 1:** NMR spectroscopic data for compounds **1** and **2**.

	1		2	
Pos.	δ_C_^a, b^	δ_H_^a, c^ (*J* in Hz)	δ_C_^a, b^	δ_H_^a, c^ (*J* in Hz)
1	67.2	3.52 (dd, 5.3, 14.3)	67.2	3.50 (dd, 5.3, 14.3)
2	72.1	3.85 (ddt, 2.4, 4.4, 9.2)	72.1	3.82 (m)
3	41.2	1.55 (m) 1.69 (m)	41.2	1.54^*^ 1.68 (dt, 4.5, 14.2)
4	71.2	3.81 (dt, 4.1, 8.2)	71.2	3.78 (m)
5	26.5	5.39 (m)	38.5	1.47 *
6	129.1	5.38 (m)	(C-6–C-12) 30.3, 30.4, 30.6, 30.7, 30.8, 30.8, 30.9	(H-6–H-12) 1.31 *
7	130.9	2.11 (m)		
8	28.2	1.34 *		
9	32.6	1.37*		
10	(C-10–C-16) 30.2, 30.4, 30.5, 30.7, 30.8, 30.8, 30.9,	(H-10–H-16) 1.36 *		
11				
12				
13			33.6	2.1 (qd, 1.6, 7.1)
14			136.2	5.71 (dt, 7.1, 15.2)
15			132.4	6.06 (dd, 10.3, 15.2)
16			138.7	6.32 (dt, 10.3, 17.0)
17	32.7	1.34 *	114.9	4.93 (dd, 1.8, 10.3) 5.07 (dd, 1.8, 17.0)
18	23.6	1.40 *		
19	14.5	0.95 (t)		

^a^ Measured in methanol-*d*_4_, ^b^ 100 MHz, ^c^ 600 MHz, * overlapped signal, assignments were done by HSQC, HMBC, COSY and NOESY.

## Data Availability

The data presented in this study are available on request from the corresponding author.
